# Proton-pump inhibitor omeprazole attenuates hyperoxia induced lung injury

**DOI:** 10.1186/s12967-016-1009-3

**Published:** 2016-08-27

**Authors:** Jute Richter, Julio Jimenez, Taro Nagatomo, Jaan Toelen, Paul Brady, Thomas Salaets, Flore Lesage, Jeroen Vanoirbeek, Jan Deprest

**Affiliations:** 1 Department of Development and Regeneration, Faculty of Medicine, KU Leuven, Leuven, Belgium; 2 Division Woman and Child, University Hospitals Leuven, Leuven, Belgium; 3 Departamento Ginecología y Obstetricia, Clínica Alemana, Santiago, Chile; 4 Department of Neonatology, Ehime Prefectural Central Hospital, Matsuyama, Japan; 5 Centre for Human Genetics, University Hospitals Leuven, KU Leuven, Leuven, Belgium; 6 Laboratory of Occupational and Environmental Toxicology, Department of Public Health and Primary Care, KU Leuven, Leuven, Belgium; 7 Clinical Department of Obstetrics and Gynaecology and Academic Department of Development and Regeneration, Organ System Cluster, University Hospitals of Leuven, Herestraat 49, 3000 Leuven, Belgium

**Keywords:** Bronchopulmonary dysplasia, Omeprazole, CYP1A1, Rabbit, Preterm

## Abstract

**Background:**

The administration of supplemental oxygen to treat ventilatory insufficiency may lead to the formation of reactive oxygen species and subsequent tissue damage. Cytochrome P4501A1 (CYP1A1) can modulate hyperoxic lung injury by a currently unknown mechanism. Our objective was to evaluate the effect of administration of omeprazole on the induction of CYP1A1 and its influence on hyperoxic lung injury in an established preterm rabbit model.

**Methods:**

Omeprazole was administered either (1) directly to the fetus, (2) to the mother or (3) after birth to the pups in different doses (2–10 or 20 mg/kg). Controls were injected with the same amount of saline. Pups were housed in normoxia (21 %) or hyperoxia (>95 %) for 5 days. Outcome parameters were induction of CYP1A1 measured by real-time polymerase chain reaction (RT-PCR) immediately after delivery, at day 3 and day 5 as well as lung function, morphometry and immunohistochemistry assessed at day 5 of life. Transcriptome analysis was used to define the targeted pathways.

**Results:**

Daily neonatal injections demonstrated a dose-dependent increase in CYP1A1. Lung function tests showed a significant improvement in tissue damping, tissue elasticity, total lung capacity, static compliance and elastance. Morphometry revealed a more developed lung architecture with thinned septae in animals treated with the highest dose (20 mg/kg) of omeprazole. Surfactant protein B, vascular endothelial growth factor and its receptor were significantly increased on immunohistochemical stainings after omeprazole treatment.

**Conclusions:**

Neonatal administration of omeprazole induces CYP1A1 in a dose-dependent matter and combined pre- and postnatal administration attenuates hyperoxic lung injury in preterm rabbits, even with the lowest dose of omeprazole without clear CYP1A1 induction.

**Electronic supplementary material:**

The online version of this article (doi:10.1186/s12967-016-1009-3) contains supplementary material, which is available to authorized users.

## Background

Although the incidence of preterm birth is decreasing slightly over the last years, it still affects 12 % of pregnancies in the United States [1]. In other countries, like Belgium, the preterm delivery rate may be lower (7.6 % deliveries <37 weeks), though remains stable. Severe prematurity can affect many organ systems but respiratory insufficiency remains the major contributor to perinatal morbidity and mortality. The use of supplemental oxygen can be life-saving in preterm infants, but may also cause bronchopulmonary dysplasia (BPD). BPD is a multi-organ disorder, which affects up to 50 % of extremely low birth weight infants <1000 g [2]. Survivors with BPD are at increased risk for readmission during the 1st year of life, long-term pulmonary problems and abnormal neurodevelopmental outcome compared to those without BPD [3].

The use of excessive oxygen may lead to an increased production of reactive oxygen species (ROS) such as superoxide anion, hydroxyl radical and hydrogen peroxide [4–6] as well as the expression of pro-inflammatory cytokines [7]. ROS can oxidate or peroxide molecules like lipids, proteins, DNA or RNA and thereby changing their structure or function [8]. Hyperoxia-induced production of ROS is recognized as a major contributor to the development of BPD [9] because the tissue damage and inflammation leads to a developmental arrest of the lung.

In recent years, the importance of cytochrome P450 enzymes as well as the aryl hydrocarbon receptor (AhR) has been demonstrated in the metabolism of a number of endogenous and exogenous compounds as well as for oxygen-induced toxicity [10–13]. When a ligand binds, AhR is translocated to the nucleus, where it dimerizes with the aryl hydrocarbon nuclear translocator (ARNT). This complex can thereafter bind to aryl hydrocardon reponse elements (AhRE) like the xenobiotic response elements (XRE) or dioxin reponse elements (DRE). Those response elements function as a *cis*-acting enhancer in the regulatory domains of genes known as the AhR *gene battery*. Included in this gene battery are many phase I and phase II detoxification enzymes like CYP1A1, CYP1A2, glutathione *S*-transferase-alpha, NAD(P)H-quinone reductase-1, UDP-glucuronosyl transferase and aldehyde dehydrogenase [8]. The cytochrome P450 enzymes are a superfamily of heme-containing proteins that are involved in the metabolism of a number of endogenous and exogenous compounds [14]. CYP1A1 is typically induced by planar aromatic hydrocarbons (PAH), but hyperoxia can also induce CYP1A1 [10, 15]. The exact mechanism of induction is unknown, but the AhR seems to play an important role as AhR [10, 12, 13] or CYP1A1 [16] deficient mice react differently on hyperoxic exposure. The induction by hyperoxia decreases if it continues for over 60 h in adult mice models [4, 17].

It has been suggested that CYP1A1 may modulate hyperoxic lung injury by scavenging of the ROS. Possible inducers besides PAH are beta-naphthoflavone (BNF) or 3-methylcholanthrene (3-MC). Also omeprazole (OM) induces CYP1A1 possibly through an AhR-mediated process. Omeprazole does not have the structural features of a typical AhR-ligand [18] and does not seem to bind AhR in a classical way [19, 20]. Possible explanations of transcription of CYP1A1 after administration of omeprazole are the decrease of the interaction forces which keep the AhR complex in a silencing state and thus a transformation of the AhR into a DNA binding form [19]. A nuclear accumulation of a DNA-binding form of the AhR activates CYP1A1 transcription [19]. Also, omeprazole seems to activate CYP1A1 transcription through common regulatory regions as typical AhR ligands [21].

Omeprazole is a proton-pump inhibitor, which is widely used to treat gastro-oesophageal reflux disease or gastric ulcers by inhibition of gastric acid secretion in both humans and animals. It is plasma-protein bound for 97 % and is metabolized through the CYP2C19 and CYP1A4. The half-life is less than 1 h in humans, and elimination occurs renal for 80 %. The drug is approved for administration to infants and neonates as well as pregnant women [22]. Transplacental transfer is present but low, and dependent on the maternal plasma levels [23]. To our knowledge the use of omeprazole in preventing hyperoxia induced lung injury has only been demonstrated so far in vitro [24] and in in vivo mice models [12, 25]. Herein, we test the hypothesis that in a hyperoxic induced lung injury preterm rabbit model, prenatal or neonatal administration of omeprazole (1) induces transcription of CYP1A1 in a dose-dependent way and (2) improves pulmonary outcome both on histological as well as functional level. In addition, we performed an unbiased pulmonary transcriptome analysis by RNA sequencing to assess the pathways targeted by omeprazole to explore its mechanism of action. We used our previously described model [26] of preterm rabbit pups exposed to hyperoxia to assess the induction of CYP1A1 as well as the functional outcome parameters.

## Methods

### Animal protocol

All experiments were approved by the Ethics committee for Animal Experimentation of the Faculty of Medicine of the KU Leuven. Animals were treated according to current guidelines of animal well-being. Time-mated pregnant does (hybrid of New Zealand White and Dendermonde) were obtained from the animalium of the KU Leuven. All does were housed in separate cages prior to an intervention or the cesarean section with a light–dark cycle of 12 h, a normal room temperature and free access to water and chow.

The first animals were used for a dose-finding study in which different administration routes, time points and doses of omeprazole were used to assess induction of CYP1A1 in the lung tissue. In the following animals both lung function as well as histological analysis were analyzed after pre- and postnatal administration of three different doses of omeprazole.

For the prenatal fetal injections (dose finding study), the doe was anesthetized with an intra-muscular injection consisting of a mixture of ketamin 35 mg/kg (Ketamin 1000^®^; CEVA Sante Animale, Libourne, France) and xylazine 6 mg/kg (Vexylan^®^; CEVA Sante Animale). Anesthesia was maintained using a facemask with isoflurane 1.5–2 % (Isoba^®^Vet; Abbott Laboratories Ltd., Queenborough, Kent, UK) in oxygen at 2 L/min. Does received a single subcutaneous injection with penicillin G 300.000 IU (Kela Pharma, Hoogstraten, Belgium), medroxyprogesterone acetate 0.9 mg/kg (Depo-Provera, Pharmacia Upjohn, Puurs, Belgium) and buprenorphine 0.03 mg/kg (Vetergesic, Alstoe Limited, York, UK) before the surgical procedure. Surgery was performed in sterile conditions with the rabbit positioned in a supine position on a heating pad. A midline laparotomy was used to exteriorize the uterine horns and thereafter the fetal umbilical vein was punctured with a 24 G needle (Terumo^®^ Surflo^®^ Winged IV catheters, Terumo Europe, Leuven, Belgium) under ultrasound guidance (VEVO^®^ 2100 system, Visualsonic, Toronto, Canada) to inject the fetus. To minimize the risks for miscarriage, maximum six fetuses per doe were injected.

For the maternal injections (dose finding as well as functional outcome study), the doe was anaesthetized with an intra-muscular injection of ketamine (35 mg/kg) and xylazine (6 mg/kg) where after the marginal ear vein was punctured to inject omeprazole or saline.

To deliver the pups, a cesarean section was performed at day 28 of gestation (term = 31 days) as previously described [26]. The pups used for the dose finding study at time point 0 were immediately euthanized with an intraperitoneal injection of T61^®^ before the first breath and the lungs were removed and snap frozen for determination of CYP1A1 levels. All other pups were placed in an incubator (Dräger Incubator 7310, Dräger^®^, Lübeck, Germany) at 32 °C in either normoxia (21 % O_2_) or hyperoxia (>95 % O_2_). Pups were fed twice daily with a mixture of special formula milk containing 30 % of proteins and 50 % of lipids (FoxValley 30/50, Illinois, US), Bio-Lapis for electrolytes, vitamins and probiotics (Protexin Veterinary, Somerset, UK) and Col-o-cat with a high amount of immunoglobulins (Sanobest, ‘s Hertogenbosch, Netherlands). The amount of feeding increased daily as previously described in detail [26]. Pups were daily injected with prophylactic antibiotics (penicillin and amikacine) from day 2 onwards [26]. Furthermore, daily intraperitoneal injections with placebo (=saline) or omeprazole were performed.

Pups were harvested immediately after delivery, at day 3 and 5 for the dose finding study, and at day 5 for the functional analysis.

## Experimental groups

### Dose finding study

Fifteen does (65 pups) were used for pre- or postnatal maternal, fetal and neonatal injections of omeprazole to assess the level of induction of CYP1A1 (Table 1).

- Fetal injections: fetuses were administered omeprazole into the umbilical vein under ultrasound guidance at 2 different time points (day 26 or day 27 of gestation, = 24 or 48 h prior to delivery) and with 2 different doses of omeprazole (low = 2 mg/kg or high = 20 mg/kg). The dose was calculated with an estimated fetal weight described by our group previously [27]. This generated four groups, with a minimum of six pups per group (−48 h low/−48 h high/−24 h low/−24 h high). All injected pups were harvested by cesarean section at day 28 of gestation for immediate preservation of lung tissue.
- Prenatal maternal injections: under anesthesia other does were injected with omeprazole (low dose: 2 mg/kg) in the marginal ear vein 48, 24 or 8 h prior to delivery. All their pups (minimum 7) were harvested immediately after delivery.
- Neonatal injections: Pups held in hyperoxia were once daily injected intraperitoneally with one of three different doses of omeprazole (low: 2 mg/kg; medium: 10 mg/kg; high: 20 mg/kg) starting immediately after delivery. Lungs were harvested at day 3 and 5. Pups housed in normoxia were only injected with the highest dose of omeprazole (=20 mg/kg/day).

Control animals were injected with a similar amount of saline and harvested at the same time points as treated animals. Untouched animals harvested immediately after delivery were used as the control group normalized to 1.

**Table 1:** Dose finding study

Administration	Injections						Harvest
Fetal	−48 h saline	−48 h OM low	−48 h OM high	−24 h saline	−24 h OM low	−24 h OM high	At delivery
Maternal	−48 h saline	−48 h OM low	−24 h saline	−24 h OMlow	−8 h saline	−8 h OM low	At delivery
Neonatal	Daily saline (normoxia)	Daily OM high (normoxia)	Daily saline (hyperoxia)	Daily OM low (hyperoxia)	Daily OM med (hyperoxia)	Daily OM high (hypreoxia)	Day 3 –day 5

### Functional assessment

Fifteen does (96 pups) were used for the assessment of lung function and histology (morphometry and immunohistochemistry) at day 5 of life Omeprazole was administered prenatally to the doe (2 mg/kg) in combination with daily neonatal intraperitoneal injections (low: 2 mg/kg, medium 10 mg/kg and high 20 mg/kg) to the pups held in hyperoxia. Control animals were injected the same amount of saline. This lead to the following groups: (1) saline-injected, housed in normoxia (normo-saline); (2) saline-injected, housed in hyperoxia (hyper-saline); (3) low dose of omeprazole, housed in hyperoxia (hyper-OMLow); (4) medium dose of omeprazole, housed in hyperoxia (hyper-OMmed) and finally (5) high dose of omeprazole, housed in hyperoxia (hyper-OMhigh). Survival of the pups was assessed on a daily basis.

### Quantification of CYP1A1 expression

Total RNA from freshly harvested and snap frozen lungs was isolated using TriPure Isolation Reagent (Roche Diagnostics, Vilvoorde, Belgium) according to the manufacturer’s guidelines. A maximum of 100 mg tissue was used per sample. The concentration was evaluated with NanoDrop ND-10000 spectrophotometer (NanoDrop Technologies, Wilmington, US). RNA was reversed transcribed to cDNA using TaqMan Reverse Transcription Reagents (Applied Biosystems, Gent, Belgium). Real-time quantitative PCR analysis was performed with an ABI Prism 7000 detection system (Applied Biosystems) using Platinum SYBR Green qPCR Supermix-UDG (Invitrogen Life Technologies, Gent, Belgium). Primers were obtained from Integrated DNA Technologies (IDT, Heverlee, Beglium) and Actin beta (ActB) was used as a housekeeping gene to normalize mRNA levels. The following primer sequences were: FWD 5′- GCACCGCAAGTGCTTCTA -3′ and REV 5′- GCCAATCTCGTCTCGTTTCT -3′ for ActB and FWD 5′- CATCTGTGCCATGTGCTTTG -3′ and REV 5′- TAGCGGAGGATGAGGAAGAA -3′ for CYP1A1. The relative mRNA levels for CYP1A1 were normalized to their ActB content. The ΔΔC_t_ method was used to calculate the fold change in mRNA expression, where ΔC_t_ = C_t(CYP1A1 gene_)−C_t(ActB gene)_ and ΔΔC_t_ = ΔC_t (Omeprazole_)−ΔC_t(saline)_; fold change = 2^(−ΔΔCt)^.

### Lung function testing

Pups were anesthetized with ketamine (35 mg/kg) and xylazin (6 mg/kg) as previously described [26]. A FlexiVent analysis [28], an invasive measurement which is the gold standard for lung function testing in vivo, was performed on anesthetized pups and the following parameters were assessed: airway resistance (Rn), tissue damping (resistance, G) and tissue elasticity (H) using Primewave-8 forced oscillation and the total lung capacity (A), static compliance (Cst) and static elastance (Est) using the pressure–volume perturbation. All measurements were performed until three consistent measurements were obtained, with a coefficient of determination of >0.95 as the limit to accept the measurement. The average of these three measurements was calculated for further reporting. After the measurements, pups were euthanized using an intracardiac injection of 0.1 mL of T61^®^.

### Morphometry

After lung function assessment, pups were euthanized, a thoracotomy was performed to remove the lungs and trachea ‘en bloc’. The left bronchus was ligated following which the left lung was removed and snap frozen for determination of CYP1A1 levels. A 20G catheter was inserted into the trachea to pressure-fix the right lung with 4 % paraformaldehyde by immersion and a constant hydrostatic pressure of 25 cm H_2_O for 24 h. After embedding, 5 µm paraffin sections were stained with hematoxylin and eosin (HE). Morphometric measurements consisted of (1) the linear intercept (Lm), which is a measure of alveolar size, (2) the mean terminal bronchiolar density (MTBD) which is inversely correlated to the number of alveoli supplied by each bronchiole and finally (3) the mean wall transection length (Lmw), a measure of the interalveolar septal thickness [29]. Vascular morphometry was performed on sections stained with Miller’s elastic staining of lungs harvested in pups held in normoxia, or in hyperoxia treated with saline or OMhigh. The external diameter and internal diameter were measured along the shortest axis of peripheral muscularized vessels with less than 100 µm external diameter. These parameters were used to calculate the proportionate medial thickness (% MT = ED−ID/ED × 100) as previously described [29].

### Immunohistochemistry

Immunohistochemical staining was performed for surfactant protein B (SP-B) to determine airway maturity and for vascular endothelial growth factor (VEGF) and its receptor fetal liver kinase 1 (Flk-1) to assess vascular markers of lung maturity. Slides were incubated with (1) goat anti-mouse polyclonal anti-SP-B (Santa Cruz Biotechnology, Heidelberg, Germany), (2) mouse anti-human monoclonal anti-VEGF (NeoMarkers, Fremont, CA, US) and finally (3) mouse anti-human monoclonal anti-Flk-1 (Santa Cruz Biotechnology). Secondary antibody incubation for SP-B slides was mouse anti-goat biotin (Santa Cruz Biotechnology) plus normal rabbit serum (DakoCytomation) followed by rinsing and incubation with streptavidin alkaline phosphatase (Roche Diagnostics, Vilvoorde, Belgium), rinsing, incubation with NBT solution and counterstaining with Methyl green (DakoCytomation). After rinsing the slides stained for VEGF and Flk-1 they were incubated with peroxidase-conjugated EnVision^TM^ and reagent (DakoCytomation), rinsed with PBS, incubated with peroxidase substrate solution containing DAB, rinsed with distilled water, counterstained with hematoxylin, dehydrated and mounted. Quantification of positive cells was performed semi-automatically using ImageJ software (1.47v, NIH, Bethesda, Maryland, USA). Ten random images from each slide were processed using the Axioskop platform (Carl Zeiss, Oberkochen, Germany).

### Transcriptome analysis and validation RT-PCR

Transcriptome analysis was done on snap frozen whole left lungs of four saline- and four omeprazole-treated (high dose) rabbits held in hyperoxia. Snap frozen whole left lungs were homogenized using the TissueLyser (Qiagen) and total RNA was isolated with the RNeasy mini-kit (Qiagen), RNA concentration was measured using the Nanodrop 1000 spectrophotometer (Thermo Scientific) and RNA integrity was assessed using the RNA 6000 Nano Kit and the Bioanalyser (Agilent Technologies). mRNA isolation, cDNA conversion and sequencing library preparation was performed using the TruSeq RNA library preparation kit (Illumina). Fastq files were thereafter imported into Array Studio (Omicsoft) and mapped against the Ensembl reference rabbit genome and transcriptome (Build: Oryctolagus cuniculus.oryCun2.64). Expression values were calculated per gene and normalized to ‘reads per kilobase per million reads’ (RPKM) values as described by Mortazavi [30]. Genes with an RPKM value of <1 in all samples were excluded. RPKM values were Log transformed for downstream analysis using the general linear model function in Array Studio for group comparison. Fold changes (FC) were calculated, indicating the ratio of change in gene expression. We applied a fold change cut-off of >1.5 or <−1.5, in order to filter out non-dysregulated molecules. Due to multiple testing, false discovery rates (FDR—Benjamini-Hochberg procedure) were calculated as a measure for statistical significance of the fold change difference observed between the two groups. We considered a transcript change significant if FDR was <0.05.

Further analysis was performed using the ingenuity pathway analysis (IPA) software. The IPA ‘upstream regulator analysis’ (URA) predicts upstream regulators by combining the directional expression changes from our mRNA-sequencing, and knowledge from prior experimental reports on causal effects between molecules (endogenous and exogenous), compiled in IPA Knowledge Base. URA calculates a z-score based on the edge of dysregulation of all the downstream molecules and the uniformity of the existing evidence about the upstream–downstream relation, for every upstream regulator known to have a causal effect on at least four dysregulated transcripts. Z-scores <−2 and >2 respectively predict a significant inhibition and activation state of the upstream regulator, regardless of the actual expression level of these molecules.

The fresh frozen lung tissue of those animals was furthermore used for RT-PCR analysis to validate three genes: CA4, SCGB1A1 and VEGF. To perform the analysis, the same methodology was used as described earlier, and ActB was used as housekeeping gene. The primer sequences for the genes were: FWD 5′-GGAGTTCTCGAGCAAACTCTAC-3′ and REV 5′-CTGCGGCCTGTGACTTAAA-3′ for CA4; FW 5′-GATGCAGGGATGCAGATGAA-3′ and REV 5′-CACAGTGGGCTCTTCACTATTT-3′ for SCGB1A1 and FWD 5-ATCATGCGGATCAAACCTCA-3′ and REV 5′-CAAGGCCCACAGGGATTTTC-3′ for VEGF.

### Statistical analysis

Kaplan–Meier curves with posthoc testing were used to quantify postnatal survival of rabbit pups using GraphPad Prism 5.0 software (GraphPad, La Jolla, California, USA). For the dose finding study as well as immunohistochemical and RT-PCR analysis, a Kruskal–Wallis test followed by Dunn’s multiple comparison test for post hoc analysis was performed.

All other parameters were assessed in a regression-modeling framework, using PROC MIXED with the repeated statement in SAS (Statistical Analysis Software, Cary, USA). This method was chosen because several pups from the same doe were used, such that data are clustered, and the mother can be considered a random effect that is nested within the group. For those parameters, all three omeprazole exposed groups (low, med and high) were compared as a group against the saline treated animals held in normoxia and hyperoxia, and against each other to define significant changes. A value of p < 0.05 was considered statistically significant. All values are expressed as mean ± standard deviation.

## Results

### Omeprazole dose finding study

Direct fetal injections with omeprazole did not increase CYP1A1 expression as measured at delivery (data not shown). Increase in expression of CYP1A1 after a single maternal dose of omeprazole (2 mg/kg IV) administered 8 h prior to delivery (relative expression of CYP1A1 1.139 ± 0.4105) did not reach statistical significance compared to controls (p = 0.872).

**Fig. 1:**
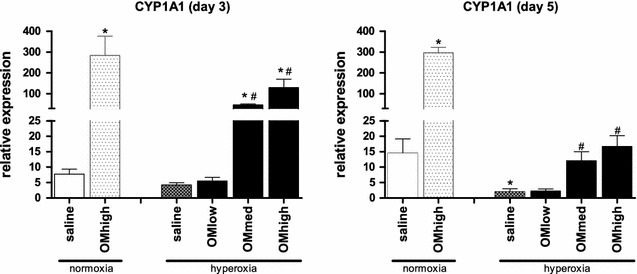
Induction of CYP1A1. Relative expression of CYP1A1 using the ΔΔCt method, measured by RT-PCR in freshly harvested lung tissue at day 3 and day 5 of life. Animals held in normoxia (injected with saline or high dose of omeprazole) or housed in hyperoxia (injected with saline or low/medium/high dose of omeprazole). *Bars* are mean ± SEM. *p < 0.05 compared to normoxia, saline treated. ^#^p < 0.05 compared to hyperoxia, saline treated

Daily neonatal injections in hyperoxia exposed pups showed a dose-dependent rise in expression of CYP1A1 (Fig. 1) both at postnatal day 3 and 5. The induction of CYP1A1 expression at day 3 was significantly higher in pups treated with the medium and high OM dose compared to saline treated animals (p < 0.001 for both). This was not the case for the low dose of omeprazole (p = 0.399). At day 5, the effect persisted, significant for the medium and high dose, yet less pronounced than on day 3 (p = 0.022 and p = 0.002 resp.). In normoxic animals, the administration of omeprazole (high dose) showed a significant induction of CYP1A1 both on day 3 and 5 (p = 0.005 and p < 0.001 resp.). Furthermore, at day 5 the CYP1A1 expression in normoxic saline-treated pups was significantly higher compared to hyperoxic saline treated pups (p = 0.027).

All further experiments were performed after a maternal injection of saline or omeprazole (2 mg/kg IV) 8 h prior to delivery, and daily neonatal injections with either saline or omeprazole (low, med, high).

### Effect of omeprazole on postnatal survival

Survival rates were 79.2 % (normo-saline), 57.6 % (hyper-saline), 67.7 % (hyper-OMlow), 65.2 % (hyper-OMmed) and 82.1 % (hyper-OMhigh). These differences did not reach statistical significance (p = 0.229).

### Omeprazole improves lung function

**Fig. 2:**
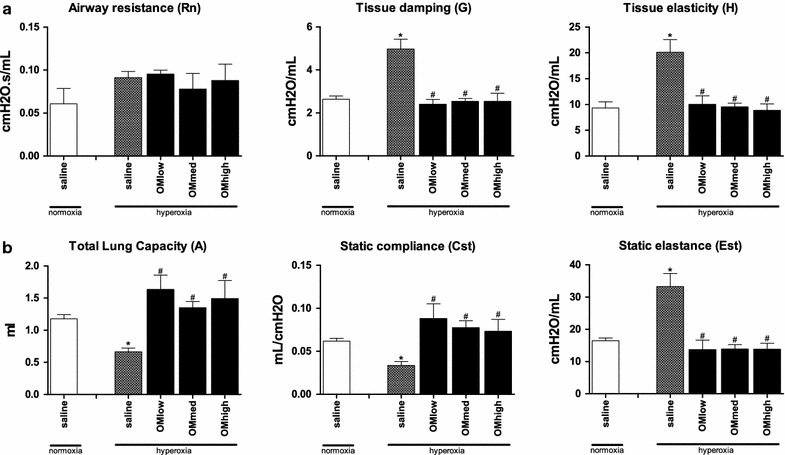
Lung function tests using forced oscillation technique. **a** Primewave-8 measurement for *Rn* airway resistance, *G* tissue damping and *H* tissue elasticity. **b** Pressure–volume perturbation for *A* total lung capacity, *Cst* static compliance and *Est* static elastance. Animals housed in normoxia (injected with saline) or hyperoxia (injected with saline or low/medium/high dose of omeprazole) and harvested day 5. *Bars* are mean ± SEM. *p < 0.05 compared to normoxia, saline treated. ^#^p < 0.05 compared to hyperoxia, saline treated

The results of the lung function tests are displayed in Fig. 2. There was no difference in the airway resistance between the different groups. Hyperoxia exposure (hyper-saline) caused a significant increase in both tissue damping and elasticity compared to normoxic controls (p = 0.0011 and p = 0.0032 resp.). Administration of variable doses of omeprazole was associated with a decreased tissue damping as well as elasticity of pups exposed to hyperoxia compared to saline-treated controls (p = 0.0007 and p = 0.0034 resp.). pressure–volume perturbation analysis revealed a significantly improved total lung capacity, static compliance and static elastance compared to saline treated animals held in hyperoxia (p < 0.001, p = 0.0001 and p = 0.0016 resp.)

### Omeprazole attenuates prematurity induced lung-developmental arrest

**Fig. 3:**
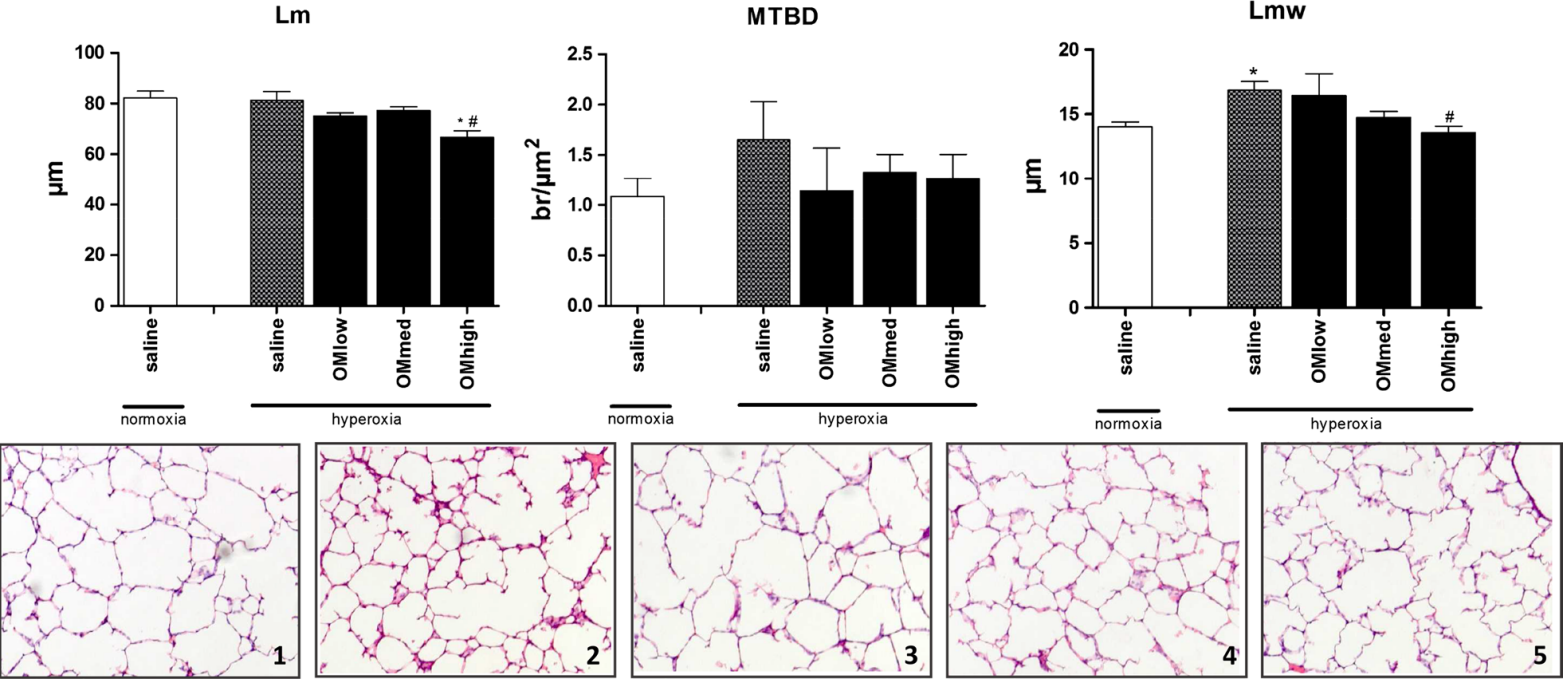
Lung morphometry. Analysis of the *Lm* linear intercept, *MTBD* mean terminal bronchiolar density and *Lmw* mean wall transection length. Animals housed in normoxia (injected with saline) or hyperoxia (injected with saline or low/medium/high dose of omeprazole) and harvested day 5. *Bars* are mean ± SEM, *1*–*5* HE staining of the right lung, animals held in normoxia (*1*), hyperoxia saline treated (*2*) or hyperoxia treated with a low (*3*), medium (*4*) or high (*5*) dose of omeprazole. *p < 0.05 compared to normoxia, saline treated. ^#^p < 0.05 compared to hyperoxia, saline treated

Morphometry results are displayed in Fig. 3. There were no significant differences between saline treated animals held in normoxia or hyperoxia for Lm (p = 0.83) neither MTBD (p = 0.23). Hyperoxia however did increase Lmw significantly (p = 0.01). Comparing all treated animals together as one group against saline treated hyperoxic animals, no significant differences were found for Lm (p = 0.08), MTBD (p = 0.34), nor Lmw (p = 0.05). Again, there were significant differences for the highest dose of OM for Lm (p = 0.02) as well as Lmw (p = 0.03). There was no obvious effect on Lm and Lmw following administration of the low or medium dose.

**Fig. 4:**
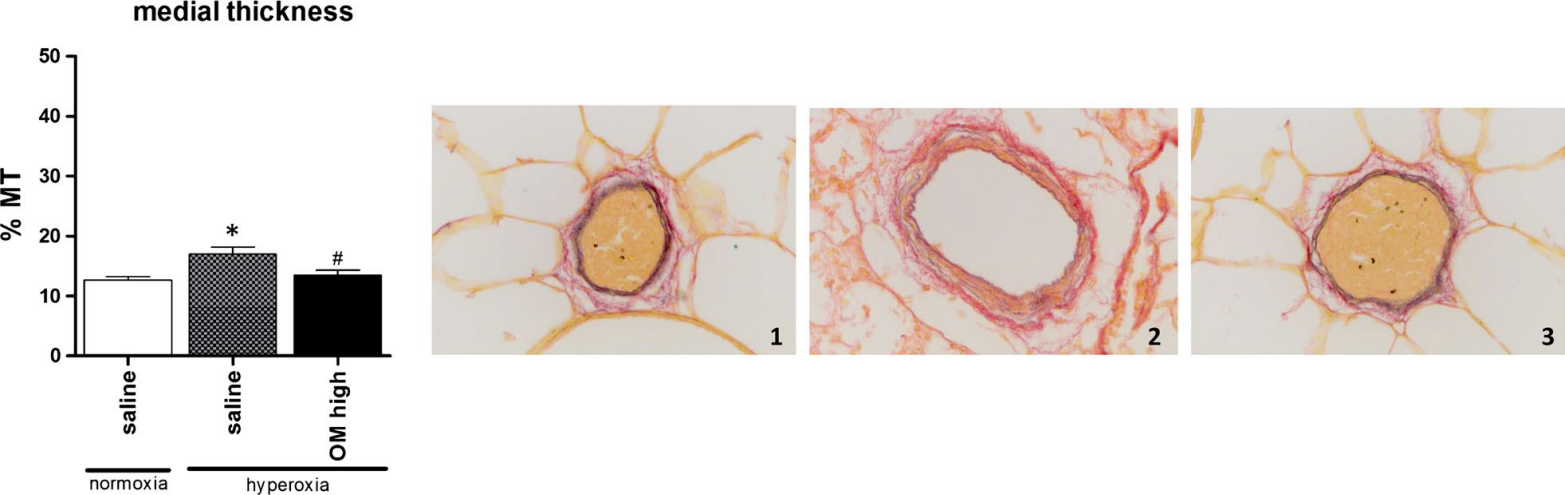
Vascular morphometry. vascular morphometry with the medial thickness (%MT) and Miller staining of a blood vessel <100 μm diameter; animals housed in normoxia (*1*), hyperoxia treated with saline (*2*) or hyperoxia treated with OM high (*3*). *p < 0.05 compared to normoxia, saline treated. ^#^p < 0.05 compared to hyperoxia, saline treated

The lungs of hyperoxic animals treated with the highest dose of omeprazole were thereafter compared with saline treated controls held in normoxia and hyperoxia. These results demonstrated a significant increase of the medial thickness of saline treated animals held in hyperoxia compared to normoxic controls. This effect was attenuated after administration of omeprazole (p = 0.007, Fig. 4).

**Fig. 5:**
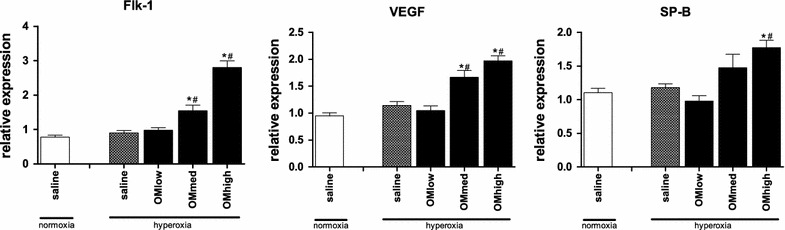
Immunohistochemstry. Immunohistochemical staining for Flk-1, VEGF and SP-B performed on lung tissue harvested day 5 of life. Animals housed in normoxia (injected with saline) or hyperoxia (injected with saline or low/medium/high dose of omeprazole) and harvested day 5. *Bars* are mean ± SEM. *p < 0.05 compared to normoxia, saline treated. ^#^p < 0.05 compared to hyperoxia, saline treated

The results of the immunohistochemical stains are shown in Fig. 5. There were no differences between both saline treated groups. Following administration of the medium and high dose of omeprazole, SP B, as well as VEGF and its receptor Flk-1 were significantly increased (p < 0.001 for all three). The lowest dose of omeprazole did not have an effect on any of the measured variables.

### Transcriptome analysis

**Fig. 6:**
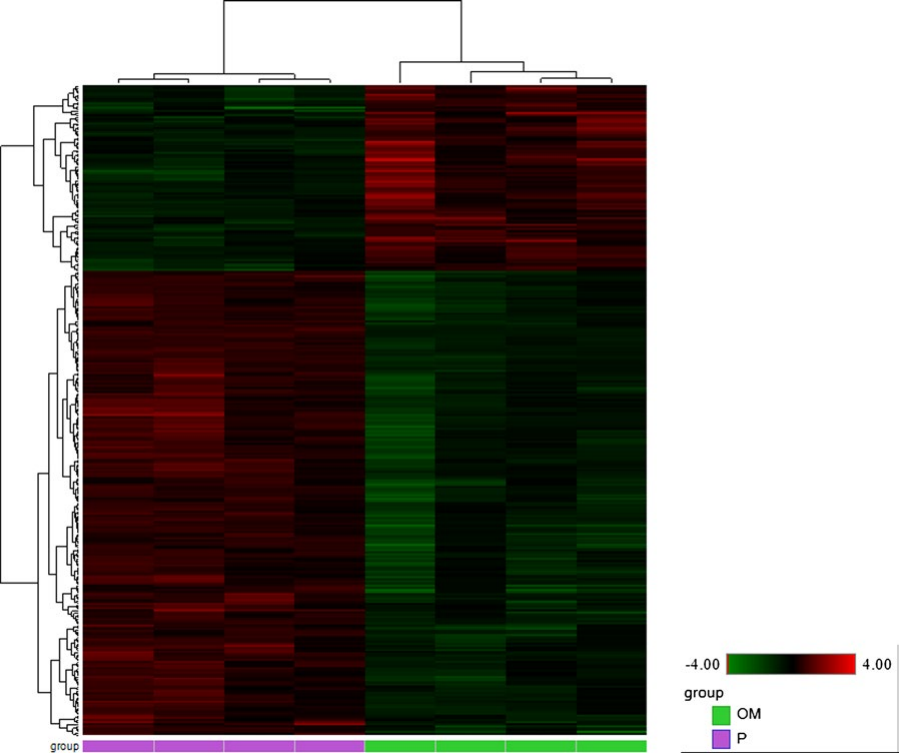
Heatmap. All transcripts with a fold change of >1.5 or <−1.5 and false discovery rate of <0.05 are shown. Color intensity displayed in the heatmap are the Log2 transformed RPKM gene expression value. These are normalised to relative low (*green*) and high (*red*) signal intensities shown in the heatmap key. *OM* omeprazole, *P* placebo

A total number of 315 transcripts were significantly dysregulated applying a filter on fold-change of >1.5 and <−1.5 with a FDR of <0.05. Expression data from all 315 dysregulated genes are displayed in a heat map (Fig. 6) where color intensity reflects the Log2 transformed RPKM gene expression values. Of these 315 transcripts, 271 had known human homologues that are recognized by IPA. Further analysis was performed using these 271 transcripts. Table 2 shows the 10 most up- and most down-regulated transcripts. The most upregulated gene in our dataset is CYP1A1 (FC 96,782; FDR 1,80 × 10^−3^), the most downregulated gene is PLEKHB2 (FC-55,950; FDR 3,79 × 10^−2^). By performing URA, we predict that 13 endogenous and exogenous molecules (Table 3) are significant upstream regulators of the transcription changes observed in our dataset.

**Table 2:** Most up- and down regulated transcripts in omeprazole treated animals

Name	Full name	FC	FDR
CYP1A1	cytochrome P450, family 1, subfamily A, polypeptide 1	96.782	1.80E−03
CA4	carbonic anhydrase IV	4.507	2.34E−02
S100A1	S100 calcium binding protein A1	3.081	2.87E−02
SCGB1A1	secretoglobin, family 1A, member 1 (uteroglobin)	2.438	3.79E−02
ASB9	ankyrin repeat and SOCS box containing 9	2.326	2.22E−02
SRGN	Serglycin	2.259	1.65E−02
PSMC3IP	PSMC3 interacting protein (HOP2)	2.087	1.64E−02
UNC13D	unc-13 homolog D (*C. elegans*)	2.059	4.59E−02
PTRHD1	peptidyl-tRNA hydrolase domain containing 1	1.967	4.00E−02
ID2	inhibitor of DNA binding 2, dominant negative helix-loop−helix protein	1.902	1.52E−02
PLEKHB2	pleckstrin homology domain containing, family B (evectins) member 2	−55.950	3.79E−02
S100A12	S100 calcium binding protein A12	−4.497	4.84E−02
CALB2	calbindin 2	−4.056	2.90E−02
KIF5C	kinesin family member 5C	−2.541	3.08E−02
ANO5	anoctamin 5	−2.428	1.10E−03
PAPPA	pregnancy-associated plasma protein A, pappalysin 1	−2.416	1.33E−02
SLC1A3	solute carrier family 1 (glial high affinity glutamate transporter), member 3	−2.352	4.97E−02
OMA1	OMA1 zinc metallopeptidase	−2.188	4.22E−02
EDA2R	ectodysplasin A2 receptor	−2.119	3.75E−02
LIMA1	LIM domain and actin binding 1	−2.034	4.94E−02

**Table 3:** Endogenous and exogenous molecules predicted to be upstream regulators of the observed transcription changes

IPA	Entrez gene name	z-score	P of overlap
Upstream regulators predicted to be activated			
miR-155-5p (miRNAs w/seed UAAUGCU)	microRNA 155-5p	2.611	2.08 × 10^−3^
miR-16-5p (and other miRNAs w/seed AGCAGCA)	microRNA 16-5p	2.425	2.58 × 10^−2^
miR-30c-5p (and other miRNAs w/seed GUAAACA)	microRNA 30c-5p	2.000	3.46 × 10^−2^
MAPK9	mitogen-activated protein kinase 9	2.189	1.18 × 10^−2^
Upstream regulators predicted to be inhibited			
VEGF	vascular endothelial growth factor (as a group)	−2.229	1.23 × 10^−3^
Gentamicin	/	−2.236	1.68 × 10^−1^
STAT3	signal transducer and activator of transcription 3	−2.613	8.60 × 10^−3^
EPAS1	endothelial PAS domain protein 1	−2.219	3.03 × 10^−3^
NKX2-3	NK2 homeobox 3	−2.091	3.65 × 10^−3^
ERG	v-ets avian erythroblastosis virus E26 oncogene homologue	−2.000	2.47 × 10^−2^
OSM	oncostatin M	−2.687	7.06 × 10^−4^
SP600125	/	−2.236	5.61 × 10^−2^
Lipopolysaccharide	/	−3.072	2.63 × 10^−2^

Based on current knowledge on the pathophysiological mechanisms involved in BPD and hyperoxia induced lung injury, and by using the analysis tools in IPA, we identified several dysregulated molecules that potentially play a role in the observed beneficiary effect of omeprazole. We demonstrate that omeprazole affects molecules involved in inflammation, reactive oxygen species (ROS) metabolism, vascular growth and development, extracellular matrix remodeling and lung development (Additional file 1: Table S1).

**Fig. 7:**
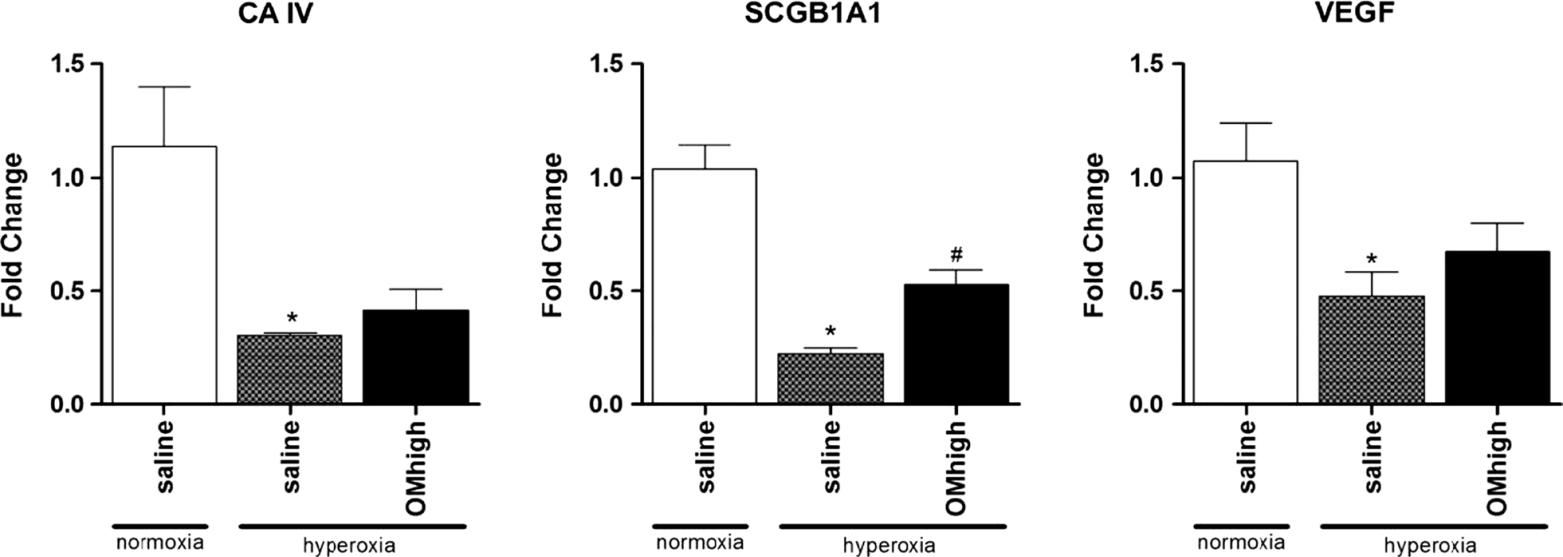
validation RT-PCR. Fold changes of CA4, SCGB1A1 and VEGF using the ΔΔCt method, measured by RT-PCR in freshly harvested lung tissue at day 5 of life. Animals held in normoxia injected. with saline, or housed in hyperoxia (injected with saline or high dose of omeprazole). *Bars* are mean ± SEM. *p < 0.05 compared to normoxia, saline treated. ^#^p < 0.05 compared to hyperoxia, saline treated

Validation RT-PCR was performed on lungs of saline-treated animals held in normoxia and hyperoxia, and animals treated with high dose of omeprazole held in hyperoxia (Fig. 7). Three genes were examined, and all three were significantly downregulated after hyperoxic exposure (p = 0.023, p < 0.001 and p = 0.038 for CA4, SCGB1A1 and VEGF resp.), but only the increase of SCGB1A1 after treatment with omeprazole reached statistical significance.

## Discussion

The principal aim of this study was to assess the effect of omeprazole treatment on hyperoxia induced lung toxicity in preterm rabbit lungs. Our central hypothesis was that omeprazole attenuates the inflammatory and functional changes in the lung tissue, potentially through the induction of the cytochrome CYP1A1. This role of CYP1A1 was demonstrated by Moorthy et al. [5] in a study where rats were pretreated with an inhibitor of CYP1A1 (1-aminobenzotriazole) with significant worsening of hyperoxic lung injury compared to controls [5]. Furthermore, recent studies have demonstrated an attenuating effect of CYP1A1 inducers on hyperoxic lung injury both in vitro [10, 24] as well as in vivo [11–13].

In the first part of our study, we analyzed the dose dependency of omeprazole induced CYP1A1 levels. Prenatal administration with the given doses, either directly (ultrasound guided fetal administration) or indirectly (maternal administration), failed to induce CYP1A1 expression in the lung tissue at birth. This might be explained by the single injection used in all prenatal administrations. Postnatal administered omeprazole efficiently and persistently (d3 and d5) increases the expression of CYP1A1 in pups exposed to normoxia. Hyperoxia has a pronounced effect on the efficiency of induction. In hyperoxia the increases were more modest and decreased over time (levels were around 45 and 5 % of what was observed in pups exposed to normoxia on day 3 and 5 resp.). This less efficient and decreasing expression could be expected, as prolonged exposure to hyperoxia has been shown to decrease the expression of CYP1A1 in adult rats [4]. Our administration dosing and regimens were based on clinical practice and literature data. In infants omeprazole is usually given at 1–2 mg/kg/day, so the low dose reflects this current clinical regimen. Rodent data about the induction of CYP1A1 by omeprazole mention higher dosages (50 mg/kg/day, [12]). Shih [31] described an obvious species specific range of induction of CYP1A1 by omeprazole.

After we established that omeprazole is an efficient CYP1A1 inducer, we set out to study the effect of omeprazole treatment on hyperoxia induced changes in preterm rabbit lungs. The exposure of rabbit lungs in the saccular stage of development to persistent high levels of oxygen (>95 %) leads to inflammatory changes and developmental arrest [26]. This becomes obvious in lung function testing by an increase in tissue damping and elasticity, a worsening in static compliance and elastance and a reduction in the total lung capacity. At the microscopic scale this corresponds to thickened septation and less developed alveoli. Omeprazole markedly attenuated all these functional changes already at the lowest dose without obvious dose dependency. Hyperoxia did not lead to changes in resistance in the conducting airways as the major pathological changes are taking place in the lung parenchyma.

This improved function would suggest a normalization of the parenchymal architecture. Indeed the mean wall transection length (Lmw), an index of the thickness of alveolar septae decreased to values that were comparable to lungs of pups kept in normoxic conditions, yet only for the highest dose of omeprazole. The distal airway complexity increased as well, evidenced by a smaller alveolar diameter (represented by Lm) but only in the cohort with the highest OM dose.

In order to analyze some possible effectors of this changes, we quantified the presence of surfactant protein B (a crude measure for lung maturation) and VEGF and one of its receptors Flk-1 (an important regulator during lung development and angiogenesis). Treatment with omeprazole had a profound dose-dependent effect on all three factors.

As the rabbit is not an easy model to study up- and downstream effects (e.g. cytokine levels, immunological changes, etc.) of oxygen toxicity due to the paucity of commercially available reagents, we also performed a transcriptome analysis. Herein we demonstrated that omeprazole affects molecules involved in inflammation, reactive oxygen species (ROS) metabolism, vascular growth and development, extracellular matrix remodeling and lung development. These processes are thought to be key features of hyperoxia induced lung injury and BPD [32]. However, apart from 1 publication on CA4 in gastric mucosa [33], CYP1A1 is the only dysregulated molecule in our dataset which has previously been related directly to omeprazole. Of course, based on our experiment, it is impossible to state whether the other observed gene changes are direct effects of omeprazole, or whether they are secondary changes due to an attenuation of the oxidative stress by e.g. CYP1A1. The results of the validation RT-PCR did demonstrate a significant increase of SCGB1A1 after omeprazole treatment, no significant changes were found for CA4 nor VEGF. Further mechanistic research needs to be performed to validate our transcriptome data, and to further elucidate the pathways involved in the beneficiary effect of omeprazole.

Other experiments already demonstrated that omeprazole modulates hyperoxia-induced lung pathology through induction of CYP1A1 [12, 24]. The importance of the CYP1A1 gene has been highlighted again in a recent study using CYP1A1 −/− mice which suggests a mechanistic role for CYP1A1 [16]. Although there was a clear dose-dependent induction of CYP1A1 after daily administrations of omeprazole, the functional outcomes had no linear correlation with CYP1A1 level, as lung function improved even at the lowest dose of omeprazole. Several explanations are possible. Firstly, several in vitro studies have demonstrated both the anti-inflammatory properties by inhibition of neutrophil function as well as anti-oxidant properties by direct scavenging activity against oxygen free radicals [34, 35]. Secondly, the induction of CYP1A1 occurs through activation of the aryl hydrocarbon receptor, as already demonstrated in multiple studies [10, 12, 13, 24]. Activation of the AhR does not only act on CYP1A1 but on a variety of molecules. The aryl hydrocarbon nuclear translocator (ARNT) is an important factor in the pathway of the hypoxia-inducible factors (HIF-1α and HIF-2α) as ARNT serves as a dimerization partner. Stabilization of HIF might lead to an increased expression of growth factors for pulmonary alveolarization and angiogenesis [36]. Furthermore, AhR is suggested to be a suppressor of lung inflammation through its interaction with nuclear factor-κB as has been shown in studies evaluating cigarette smoke [37, 38] or influenza [39]. Recently Stockinger [40] reviewed the anti-inflammatory properties of AhR activation. Lung-resident dentritic cells seem to play an important role by modulating the immune-suppressive enzyme indolamine 2, 3 dioxygenase. Furthermore, AhR activation has an important effect on the differentiation of IL-17 producing T-helper cells, and bronchial fibroblasts who are sensitive to IL-17 can produce inflammatory mediators and chemoattractants such as IL-6 and IL-8 in response to IL-17 stimulation.

A recent study performed on neonatal mice has demonstrated a potentiation of hyperoxia induced lung injury after administration of omeprazole [25]. These results are in conflict with our data, but several explanations are possible. First there is the difference in experimental animal, as Shih has demonstrated the important species difference in CYP1A1 induction [31]. Secondly, the study of Shivanna showed an increase in AhR after a short period (4 days), but this effect disappeared after 14 days of hyperoxic exposure. So there might be a time period in which omeprazole has a benefit that disappears when administered for too long with even a toxic effect depending on dose and/or duration of treatment. A more recent study from the same research group has demonstrated that there is no potentiation of hyperoxia induced cell toxicity after administration of omeprazole [41]. This might again be explained by the shorter duration of hyperoxia and administration period of omeprazole, as well as the use of human pulmonary microvascular endothelial cells instead of rodents. More research is needed to address the most effective but also safe dose and duration of treatment, before this can be translated into human care.

We acknowledge a number of limitations to our study. First, though lung development of the rabbit is closer to that of man than rodents, it still differs compared to larger animals like baboons or sheep [42]. Another limitation is the lack of data of normoxic animals treated with omeprazole, which in clinical practice would be a group not qualifying for treatment, but could have offered further insides in the effects seen after administration of omeprazole. Furthermore, the high concentration of oxygen (>95 %) and the relative short time interval of 5 days does not mimic entirely the human situation of what is referred to as the “new” BPD. In our previous model [26] we used 7 days of hyperoxic exposure, but decided for a shorter duration in an attempt to decrease mortality. We are however working to expand this model with chronic exposure to oxygen. Studies with larger animals will be needed to assess efficacy of the lowest dose, and safety of higher doses of omeprazole in the prevention of hyperoxia induced lung injury as long term exposure to omeprazole might have negative effects on outcome [25]. Another limitation might be the combined used of pre- and postnatal administration of omeprazole without assessing both independently. Possible side effects of gastric acid inhibitors used in preterm infants are the increased risk of bloodstream and respiratory infections and necrotizing enterocolitis [43] but these complications seem to occur more with the use of H_2_ antagonists rather than proton pump inhibitors like omeprazole [44].

The strengths of our study are the use of different doses of omeprazole administered through different administration routes, as well as the assessment of both lung function as well as histological parameters.

## Conclusions

Postnatal administration of omeprazole induces CYP1A1 expression in a dose-dependent manner and combined maternal and neonatal administration improves neonatal pulmonary lung function. This is paralleled by a more mature lung architecture in preterm rabbit pups exposed to hyperoxia. As the lowest dose of omeprazole had a positive effect on lung damage, without a clear increase of CYPA1A, further research will be necessary to elucidate the exact mechanism at which omeprazole attenuates the hyperoxic lung injury.

## Additional file

10.1186/s12967-016-1009-3 Molecules of special interest.

## Abbreviations

A: total lung capacity
ActB: actin beta
AhR: aryl hydrocarbon receptor
AhRE: aryl hydrocarbon response elements
BNF: beta-naphthoflavone
BPD: bronchopulmonary dysplasia
Cst: static compliance
CYP1A1: cytochrome P4501A1
DRE: dioxin response elements
Est: static elastance
FC: fold change
FDR: false discovery rate
Flk-1: fetal liver kinase 1
G: tissue damping
H: tissue elasticity
HSP: heat shock protein
IPA: ingenuity pathway analysis
Lm: linear intercept
Lmw: mean wall transection length
MTBD: mean terminal bronchiolar density
OM: omeprazole
PAH: planar aromatic hydrocarbons
Rn: airway resistance
ROS: reactive oxygen species
RPKM: reads per kilobase per million reads
RT-PCR: real-time polymerase chain reaction
SP-B: surfactant protein B
URA: upstream regulator analysis
VEGF: vascular endothelial growth factor
XRE: xenobiotic response elements
