# Supplementary material for: Proton-pump inhibitor omeprazole attenuates hyperoxia induced lung injury
Source: J Transl Med. 2016 Aug 27;14(1):247. doi: 10.1186/s12967-016-1009-3 (PMC5002203; doi:10.1186/s12967-016-1009-3)
Supplement: Supplementary file 1 — 10.1186/s12967-016-1009-3 Molecules of special interest. [file 12967_2016_1009_MOESM1_ESM.docx]

**Additional file 1: Table S1**. Molecules of special interest.

| IPA gene name | Potential role in the beneficiary effect of omeprazole on hyperoxia induced lung injury | FC | FDR | z-score |
| --- | --- | --- | --- | --- |
| ROS metabolism | | | | |
| CYP1A1 | CYP1A1 (cytochrome P450 family 1, subfamily A, polypeptide 1) is known to be induced by omeprazole (via AhR), which is associated with attenuation of hyperoxic lung injury in mice. It has been suggested that CYP1A1 is mediating this effect by detoxification of lipid peroxides and hydroperoxides generated by reactive oxygen species. [1, 2] | 96.782 | 1.80x10^-3^ |  |
| NQO1 | Induction of NQO1 (NAD(P)H dehydrogenase, quinone 1) by hyperoxia is thought to be a protective mechanism, able to scavenge and detoxify ROS-metabolites. [3] This response appears to be under the control of AhR as well. [4] | -1.612 | 3.50x10^-2^ |  |
| Inflammation | | | | |
| IL1RAP | IL1RAP (interleukin 1 receptor accessory protein) is involved in IL33-signalling. [5] The IL33-pathway has been linked to inflammatory lung diseases as COPD [6] and childhood wheezing. [7] | -1.926 | 1.85x10^-2^ |  |
| IL17RD | IL17RD (interleukin 17 receptor D) is established as a feedback antagonist of pro-inflammatory cytokines [8] and as a antagonist of FGF-signalling, likely to play critical roles in endothelial or epithelial functions such as proliferation, migration, and angiogenesis. [9] | -1.818 | 2.29x10^-2^ |  |
| SCGB1A1 | SCGB1A1 or CCSP (secretoglobin family 1A, member 1 or Clara cell secretory protein) is an immunomodulatory protein, especially expressed in Clara cells. SCGB1A1-deficient mice exhibit more severe lung damage following hyperoxia, with higher interleukin levels. [10] | 2.438 | 3.79x10^-2^ |  |
| S100A12 | S100A12 (S100 calcium binding protein A12) is granulocyte-specific marker which exerts important pro-inflammatory functions via its interaction with the receptor for advanced glycation end products. Increased levels of plasma S100A12 are associated to sepsis, … However in human BPD, no increased S100A12-levels were demonstrated. [11] | 3.081 | 2.87x10^-2^ |  |
| STAT3 | STAT3 (signal transducer and activator of transcription 3) is a transcription factor that participates in the signaling pathways for many cytokines in various cells and organs that are regulated by the suppressor of cytokine signaling (SOCS) family. This pathway has a proven role in many inflammatory lung diseases. [12] STAT3 is an essential regulator of the pulmonary response to hyperoxia. [13] |  |  | -2.613 |
| miR-155-5p | miR-155-5p (microRNA 155-5p) is, known to be involved in many inflammatory processes. [14] |  |  | 2.611 |
| Vascular growth and development | | | | |
| CA4 | CA IV (carbonic anhydrase 4) is, in the lungs, located specifically on the luminal side of the endothelium of small alveolar capillaries and it is differentially expressed in different stages of lung development. [15] | 4.507 | 2.34x10^-2^ |  |
| VEGF (group) | VEGF (vascular endothelial growth factor) is well described to be involved in angiogenesis in different tissue types. However the expression of VEGF in the lung appears to be affected by hyperoxia, the exact finalities of this response remain unclear. [16] |  |  | -2.229 |
| EPAS1 | EPAS1 (endothelial PAS domain protein 1) is a hypoxia-responsive transcription factor with relatively high expression levels in the lung and in endothelial cells. It was recently proposed as a key regulator of chronic obstructive pulmonary disease in adults. [17] An activated mutation in EPAS1 has also been associated to pulmonary hypertension. [18] |  |  | -2.219 |
| ERG | ERG (v-ets avian erythroblastosis virus E26 oncogene homologue) is an endothelial transcription factor and is essential to maintain vascular stability. [19] |  |  | -2.000 |
| Connective tissue and extracellular matrix remodeling | | | | |
| BMP2 | BMP2 (bone morphogenetic protein 2) is a member of the BMP-signaling pathway. Smad 1/5/9 (Smad family member 1/5/9) are downstream mediators of BMP-signaling, that act as transcription regulators. BMP2 is upregulated after epithelial cell injury and causes epithelial dysfunction and hyperpermeability mediated by Smad 1/5-dependent downregulation of E-cadherin. [20] This pathway is also involved in pulmonary vascular homeostasis and dysregulations are known to cause pulmonary hypertension. [21] BMP-signaling is active during late lung development. [22] | -1.623 | 3.03x10^-2^ |  |
| SMAD5 |  | -1.505 | 1.62x10^-2^ |  |
| SMAD9 |  | -1.561 | 3.29x10^-2^ |  |
| ITGA1 | Integrins contribute to the regulation of extracellular matrix composition, in addition to supplying in some tissues a proliferative and survival signal. It also provides a tissue retention function for cells of the immune system, … In several disease models integrins (amongst others α1, αv, β6) were shown to be involved in airway remodeling, tissue fibrosis, alveolar and vascular permeability and also inflammation. [23-26] | -1.959 | 1.85x10^-2^ |  |
| ITGAV |  | -1.817 | 1.85x10^-2^ |  |
| ITGB6 |  | -1.735 | 4.19x10^-2^ |  |
| OSM | OSM (oncostatin M) has been shown to increase collagen deposition, in a STAT3 and Smad 1/5/8-dependent way, when overexpressed in a mouse model of lung fibrosis. [27] It also has a chemotactic influence on neutrophiles in mice with pneumonia [28] and suppresses lung development in fetal rats. [29] |  |  | -2.687 |
| Lung development | | | | |
| ID2 | ID2 (inhibitor of DNA binding 2) marks distal tip multipotent progenitor cells, during branching morphogenesis (and later stages of lung development). [30] It is involved in BMP-signaling as a downstream effector molecule, and is induced in the saccular and alveolar stage of lung development. [22] ID2 also antagonizes the TGFβ1-dependent fibrogenic activity of pulmonary myofibroblasts. [31] | 1.902 | 1.52x10^-2^ |  |

1. Shivanna, B., et al., *Omeprazole attenuates hyperoxic lung injury in mice via aryl hydrocarbon receptor activation and is associated with increased expression of cytochrome P4501A enzymes.* J Pharmacol Exp Ther, 2011. **339**(1): p. 106-14.

2. Lingappan, K., et al., *Mice deficient in the gene for cytochrome P450 (CYP)1A1 are more susceptible than wild-type to hyperoxic lung injury: evidence for protective role of CYP1A1 against oxidative stress.* Toxicol Sci, 2014. **141**(1): p. 68-77.

3. Merker, M.P., et al., *Influence of pulmonary arterial endothelial cells on quinone redox status: effect of hyperoxia-induced NAD(P)H:quinone oxidoreductase 1.* Am J Physiol Lung Cell Mol Physiol, 2006. **290**(3): p. L607-19.

4. Zhang, S., et al., *Aryl hydrocarbon receptor is necessary to protect fetal human pulmonary microvascular endothelial cells against hyperoxic injury: Mechanistic roles of antioxidant enzymes and RelB.* Toxicol Appl Pharmacol, 2015. **286**(2): p. 92-101.

5. Martin, M.U., *Special aspects of interleukin-33 and the IL-33 receptor complex.* Semin Immunol, 2013. **25**(6): p. 449-57.

6. Xia, J., et al., *Increased IL-33 expression in chronic obstructive pulmonary disease.* Am J Physiol Lung Cell Mol Physiol, 2015. **308**(7): p. L619-27.

7. Savenije, O.E., et al., *Association of IL33-IL-1 receptor-like 1 (IL1RL1) pathway polymorphisms with wheezing phenotypes and asthma in childhood.* J Allergy Clin Immunol, 2014. **134**(1): p. 170-7.

8. Fuchs, Y., et al., *Sef is an inhibitor of proinflammatory cytokine signaling, acting by cytoplasmic sequestration of NF-kappaB.* Dev Cell, 2012. **23**(3): p. 611-23.

9. Yang, R.B., et al., *A novel interleukin-17 receptor-like protein identified in human umbilical vein endothelial cells antagonizes basic fibroblast growth factor-induced signaling.* J Biol Chem, 2003. **278**(35): p. 33232-8.

10. Johnston, C.J., et al., *Altered pulmonary response to hyperoxia in Clara cell secretory protein deficient mice.* Am J Respir Cell Mol Biol, 1997. **17**(2): p. 147-55.

11. Loughran-Fowlds, A., et al., *Respiratory disease and early serum S100A12 changes in very premature infants.* Acta Paediatr, 2011. **100**(12): p. 1538-43.

12. Gao, H. and P.A. Ward, *STAT3 and suppressor of cytokine signaling 3: potential targets in lung inflammatory responses.* Expert Opin Ther Targets, 2007. **11**(7): p. 869-80.

13. Hokuto, I., et al., *Stat-3 is required for pulmonary homeostasis during hyperoxia.* J Clin Invest, 2004. **113**(1): p. 28-37.

14. Elton, T.S., et al., *Regulation of the MIR155 host gene in physiological and pathological processes.* Gene, 2013. **532**(1): p. 1-12.

15. Fleming, R.E., et al., *Pulmonary carbonic anhydrase IV: developmental regulation and cell-specific expression in the capillary endothelium.* Am J Physiol, 1993. **265**(6 Pt 1): p. L627-35.

16. Meller, S. and V. Bhandari, *VEGF levels in humans and animal models with RDS and BPD: temporal relationships.* Exp Lung Res, 2012. **38**(4): p. 192-203.

17. Yoo, S., et al., *Integrative analysis of DNA methylation and gene expression data identifies EPAS1 as a key regulator of COPD.* PLoS Genet, 2015. **11**(1): p. e1004898.

18. Gale, D.P., et al., *Autosomal dominant erythrocytosis and pulmonary arterial hypertension associated with an activating HIF2 alpha mutation*, in *Blood*. 2008: United States. p. 919-21.

19. Birdsey, G.M., et al., *The endothelial transcription factor ERG promotes vascular stability and growth through Wnt/beta-catenin signaling.* Dev Cell, 2015. **32**(1): p. 82-96.

20. Helbing, T., et al., *Inhibition of BMP activity protects epithelial barrier function in lung injury.* J Pathol, 2013. **231**(1): p. 105-16.

21. Upton, P.D. and N.W. Morrell, *The transforming growth factor-beta-bone morphogenetic protein type signalling pathway in pulmonary vascular homeostasis and disease.* Exp Physiol, 2013. **98**(8): p. 1262-6.

22. Alejandre-Alcazar, M.A., et al., *Temporal and spatial regulation of bone morphogenetic protein signaling in late lung development.* Dev Dyn, 2007. **236**(10): p. 2825-35.

23. Wright, D.B., H. Meurs, and B.G. Dekkers, *Integrins: therapeutic targets in airway hyperresponsiveness and remodelling?* Trends Pharmacol Sci, 2014. **35**(11): p. 567-74.

24. Gardner, H., *Integrin alpha1beta1.* Adv Exp Med Biol, 2014. **819**: p. 21-39.

25. Madala, S.K., et al., *Inhibition of the alphavbeta6 integrin leads to limited alteration of TGF-alpha-induced pulmonary fibrosis.* Am J Physiol Lung Cell Mol Physiol, 2014. **306**(8): p. L726-35.

26. Sheppard, D., *Modulation of acute lung injury by integrins.* Proc Am Thorac Soc, 2012. **9**(3): p. 126-9.

27. Wong, S., et al., *Oncostatin M overexpression induces matrix deposition, STAT3 activation, and SMAD1 Dysregulation in lungs of fibrosis-resistant BALB/c mice.* Lab Invest, 2014. **94**(9): p. 1003-16.

28. Traber, K.E., et al., *Oncostatin M Induces STAT3-dependent CXCL5 Expression and Neutrophil Recruitment During Pneumonia.* Am J Respir Cell Mol Biol, 2015.

29. Nogueira-Silva, C., et al., *The role of glycoprotein 130 family of cytokines in fetal rat lung development.* PLoS One, 2013. **8**(6): p. e67607.

30. Rawlins, E.L., et al., *The Id2+ distal tip lung epithelium contains individual multipotent embryonic progenitor cells.* Development, 2009. **136**(22): p. 3741-5.

31. Izumi, N., et al., *BMP-7 opposes TGF-beta1-mediated collagen induction in mouse pulmonary myofibroblasts through Id2.* Am J Physiol Lung Cell Mol Physiol, 2006. **290**(1): p. L120-6.
